# Impact of Poorly Controlled Diabetes and Glycosylated Hemoglobin Values in Geriatric Hip Fracture Mortality Risk Assessment

**DOI:** 10.7759/cureus.36422

**Published:** 2023-03-20

**Authors:** Lauren A Merrell, Garrett W Esper, Abhishek Ganta, Kenneth A Egol, Sanjit R Konda

**Affiliations:** 1 Orthopedic Surgery, New York University (NYU) Langone Orthopedic Hospital, New York, USA; 2 Orthopedic Surgery, State University of New York (SUNY) Upstate Medical University, Syracuse, USA; 3 Orthopedic Surgery, New York University (NYU) Langone Health, New York, USA; 4 Orthopedic Surgery, Jamaica Hospital Medical Center, New York, USA; 5 Orthopaedic Surgery, Jamaica Hospital Medical Center, New York, USA

**Keywords:** risk stratification, geriatric, hip fracture, hemoglobin a1c, diabetes

## Abstract

Introduction

The presence of poorly-controlled diabetes in the setting of geriatric hip fractures has been shown to increase all-cause mortality and worsen outcomes. This study aimed to assess whether the addition of a patient's glycated hemoglobin (A1c) value to a validated geriatric inpatient risk tool improves the predictive capacity of the risk tool.

Methods

A cohort of 2430 patients >55 years old treated for low-energy mechanism hip fractures between October 2014 to November 2021 were reviewed for demographics (including diabetes diagnoses and their respective hemoglobin A1c values at the time of admission), injury details, hospital quality measures, and mortality. As past work demonstrated a hemoglobin A1c value above 8% to be the tipping point for worse outcomes, the baseline Score for Trauma Triage in the Geriatric and Middle-Aged (STTGMA) tool for inpatient mortality in hip fractures (STTGMAHIP_FX_SCORE - Score for Trauma Triage in the Geriatric and Middle-Aged Hip Fracture Score) was modified to include a patient's hemoglobin A1c using an 8% cutoff (STTGMAHIP_8%A1c - Score for Trauma Triage in the Geriatric and Middle-Aged Hip 8% Hemoglobin A1c Cutoff Score). The new model's predictive ability (as measured by the area under the receiver operating curves (AUROCs)) for inpatient mortality was compared to the baseline tool using DeLong's test. Risk quartiles were generated for the new tool, and comparative analyses were conducted on hospital quality measures and outcomes.

Results

Five hundred and sixty-five patients (23%) were noted to have diabetes mellitus, and 76 patients had an A1c above 8%. Patients with a hemoglobin A1c above 8% had a higher rate of inpatient complications and mortality through one year. The STTGMAHIP_8%A1c score significantly improved the predictive capacity for inpatient mortality compared to STTGMAHIP_FX_SCORE (0.786 vs. 0.672, p=0.0456). Upon analysis of the risk quartiles, the highest risk cohort was found to have a longer length of stay (p<0.001), with higher rates of inpatient (p<0.001) and 30-day mortality (p<0.001) and need for admission to the intensive care unit (p<0.001) as compared to the minimal risk cohort. Patients in the lowest risk quartile were most likely to be discharged home (p<0.001).

Conclusion

Patients who present with a hemoglobin A1c above 8% experienced significantly worse outcomes than those below 8%. The inclusion of a patient's hemoglobin A1c as a cutoff score improves the STTGMAHIP_FX_SCORE tool to predict mortality and risk stratify patient outcomes. While diabetes presents another medical challenge to manage, providers may utilize this new variable to better highlight at-risk diabetic patients.

## Introduction

Individuals who sustain a hip fracture are most often elderly and have a host of medical comorbidities that increase their risk of morbidity and mortality [1]. One particular comorbidity that has been associated with worse outcomes following hip fracture is diabetes mellitus [2].

The literature highlights the independent association between a diagnosis of diabetes mellitus and poor outcomes following hip fracture. A study by Spaetgens et al. controlled for other confounding factors, and while patients with diabetes often had more severe fractures, their analysis still found a 1.7x higher absolute mortality rate associated with diabetes [2]. In addition to a patient's additional medical history, the level of glycemic control found in diabetic patients is also an important variable to consider. While point-of-care glucose readings can provide information to providers, studies have demonstrated stress-induced hyperglycemia following hip fractures and other traumatic injuries [3]. As a result, glucose readings around the time of injury may not be accurate to a patient's normal baseline glycemic control. Therefore, the usage of glycosylated hemoglobin, or hemoglobin A1c (HgA1c), is a practical and effective alternative to assess long-term glycemic control [4]. Studies have also shown a correlation between elevated hemoglobin A1c values and poor outcomes following trauma, further highlighting the utility of this lab test [5,6]. In particular, a HgA1c value above 8% has been shown to be the inflection point at which the risk of complications and mortality starts to increase compared to nondiabetic patients (unpublished journal article: Merrell LA, Esper GW, Gibbons K, Ganta A, Egol KA, Konda SR. Poorly Controlled Diabetes: Glycosylated Hemoglobin (HA1c) Levels >8% are the Tipping Point for Significantly Worse Outcomes Following Hip Fracture in the Geriatric Population, 3/4/23; further referred to as Merrell et al.).

As patients who sustain a hip fracture face high rates of morbidity and mortality from the injury and recovery alone, it is important to investigate methods to account for additional risk factors that can either be modified to reduce risk or at least add prognostic value to improve short and long-term care coordination. A validated inpatient mortality risk assessment tool, the Score for Trauma Triage in the Geriatric and Middle-Aged (STTGMA), demonstrates one method to accomplish this goal. This tool constructs a score for all middle-aged and geriatric patients 55 and older who sustain different orthopedic trauma injuries in order to modulate their care levels and trajectory based on effective risk stratification [7]. As the original STTGMA tool utilized clinical data that is readily available on arrival to the emergency department, this study group continues to research additional accessible variables that may further improve our predictive capacity and refine the tool's efficacy. The original STTGMA tool included a patient's age and injury details, including the Abbreviated Injury Score (AIS) for the head/neck and chest, Glasgow Coma Scale (GCS), and comorbidity profile as defined by the Charlson Comorbidity Index (CCI) before additional research highlighted new variables such as a patient's baseline ambulatory status, American Society of Anesthesiologists (ASA) score, their COVID-19 status on hospital admission, baseline body mass index, smoking status at the time of injury, the season in which they sustain their injury [8-12]. More recently, STTGMA has become more adaptive to a patient's changing risk profile throughout their admission, now accounting for the occurrence of inpatient complications [13]. As previous studies have shown the importance and utility of including new variables to the score, the literature showing an association between hemoglobin A1c levels and mortality represents another possible variable to examine. 

This study aims to assess whether the addition of a patient's hemoglobin A1c value above or below an 8% cutoff at the time of admission to the inpatient STTGMA tool improves the predictive capacity for mortality in the middle-aged and geriatric hip fracture populations.

## Materials and methods

This retrospective cohort study utilized a prospectively collected hip fracture database (approved by the New York University (NYU) Langone Health Office of Science and Research Institutional Review Board, study number i20-01766) to review all patients who were aged 55 and older and sustained a hip fracture through a low-energy mechanism between October 2014 to November 2021. This study's criteria for a low-energy mechanism included a fall from standing or from a height of less than two stairs. All patients included in the study were treated at one academic medical center following a subtrochanteric, femoral neck, or intertrochanteric hip fracture. All fractures were classified according to the AO Foundation/Orthopaedic Trauma Association (AO/OTA) fracture classifications: 31A, 31B, 32(A-C). Patients were excluded if they sustained their injury via a high-energy mechanism or were younger than 55 years old.

Each patient included in the analysis was reviewed for demographics that included age, sex, and comorbidities as represented by the Charlson Comorbidity Index (CCI), body mass index (BMI), and baseline ambulatory status. All patients were also reviewed for diabetes diagnoses and their respective hemoglobin A1c values at the time of admission. Injury presentation variables collected were GCS and Abbreviated Injury Score (AIS) for both the head/neck (AIS H/N) and chest (AIS C).

Hospital quality measures collected were the length of stay (LOS) in days, the need for admission to the intensive care unit (ICU), and the rate of discharge home (for this study, a discharge home was defined as either discharge to a patient's home independently or with a health service). Mortality measures collected included inpatient, within 30 days, and within one year of hospital discharge. The incidences of inpatient complications during each patient's admission were recorded and divided into major and minor complications. Major complications included sepsis/septic shock, pneumonia, deep vein thrombus/pulmonary embolism (DVT/PE), myocardial infarction (MI), stroke, acute respiratory failure (ARF), and cardiac arrest. Minor complications included the incidence of surgical site infection (SSI), acute renal failure/acute kidney injury (AKI), urinary tract infection (UTI), decubitus ulcer, and acute blood loss anemia.

Patients with a past medical history of diagnosed diabetes were reviewed for their laboratory values on the presentation for their injuries. An initial comparative analysis conducted in another study by this research group determined the hemoglobin A1c cutoff value at which patient outcomes significantly worsened (Merrell et al.). As this was identified to be an A1c value of 8%, this cutoff was used to convert each patient's A1c value into a binary variable as above 8% or below 8%.

The baseline STTGMA score for hip fractures (STTGMAHIP_FX_SCORE - Score for Trauma Triage in the Geriatric and Middle-Aged Hip Fracture Score) was calculated for each patient. The predictive model was then adapted to include a patient's A1c value utilizing the specified A1c cutoff of 8% (STTGMAHIP_8%A1c - Score for Trauma Triage in the Geriatric and Middle-Aged Hip 8% Hemoglobin A1c Cutoff Score). Next, the predictive ability of each model was compared using DeLong's test to assess each score's respective area under the receiver operating curves (AUROCs). Next, patients were stratified into risk quartiles based on their new respective STTGMAHIP_8%A1c mortality risk scores. Comparative analyses of the hospital quality measures and outcomes of each risk quartile were conducted to confirm the efficacy of the new dichotomous A1c variable as a predictive factor.

The following statistical tests were used as appropriate: Chi-square tests for categorical variables, Mann-Whitney U tests, independent sample t-tests, and analyses of variance (ANOVA) for continuous or linear variables. All statistics were calculated with IBM SPSS data software, version 25 (IBM Inc., Armonk, USA). An alpha of 0.05 was used as a threshold for significance to compare AUROCs utilizing DeLong's test. As our comparative analysis between quartiles assessed multiple different variables, a Bonferroni adjusted alpha (0.05/10=0.005) was used as the threshold for significance in our quartile analysis [14].

## Results

Our overall cohort included 2430 patients that met inclusion criteria. The demographic characteristics for the overall cohort included the following breakdown: the majority of patients were White (71%), female (69%), with a mean age of 80.7 years ± 10.2 and mean BMI of 24.17 ± 4.94 kg/m^2^. Most patients were community ambulators (68%) at baseline, while 28% were household ambulators, and 3.92% were non-ambulatory. The majority of patients sustained an intertrochanteric (31A, 53%) or femoral neck (31B, 43%) fracture (Table 1). 

**Table 1:** Overall cohort demographics AIS - Abbreviated Injury Score; CRPP - closed reduction percutaneous pinning

Demographics	Total n (%)
N	2430
Variables	
Age (years; mean ± SD)	80.71 ± 10.20
Body Mass Index	24.17 ± 4.93
Charlson Comorbidity Index	1.51 ± 1.72
Gender	
Male	743 (30.58%)
Female	1687 (69.42%)
Race	
White	1728 (71.11%)
Black	180 (7.41%)
Hispanic	124 (5.10%)
Asian	200 (8.23%)
Other	44 (1.81%)
Unknown	113 (4.65%)
Ambulatory status	
Community ambulator	1649 (67.86%)
Household ambulator	685 (28.19%)
Non-ambulatory/wheelchair	95 (3.91%)
Glasgow Coma Scale (mean ± SD)	14.87 ± 0.63
AIS head/neck (mean ± SD)	0.03 ± 0.27
AIS chest (mean ± SD)	0.02 ± 0.19
Fracture classification	
31A	1282 (52.76%)
31B	1051 (43.25%)
32A	63 (2.59%)
32B	3 (0.12%)
32C	31 (1.28%)
Treatment	
Short cephalomedullary nail	974 (40.08%)
Long cephalomedullary nail	336 (13.83%)
Hemiarthroplasty	560 (23.05%)
Total hip arthroplasty	153 (6.30%)
Sliding hip screw	101 (4.16%)
CRPP	210 (8.64%)
Non-operative	96 (3.95%)

**Figure 1:**
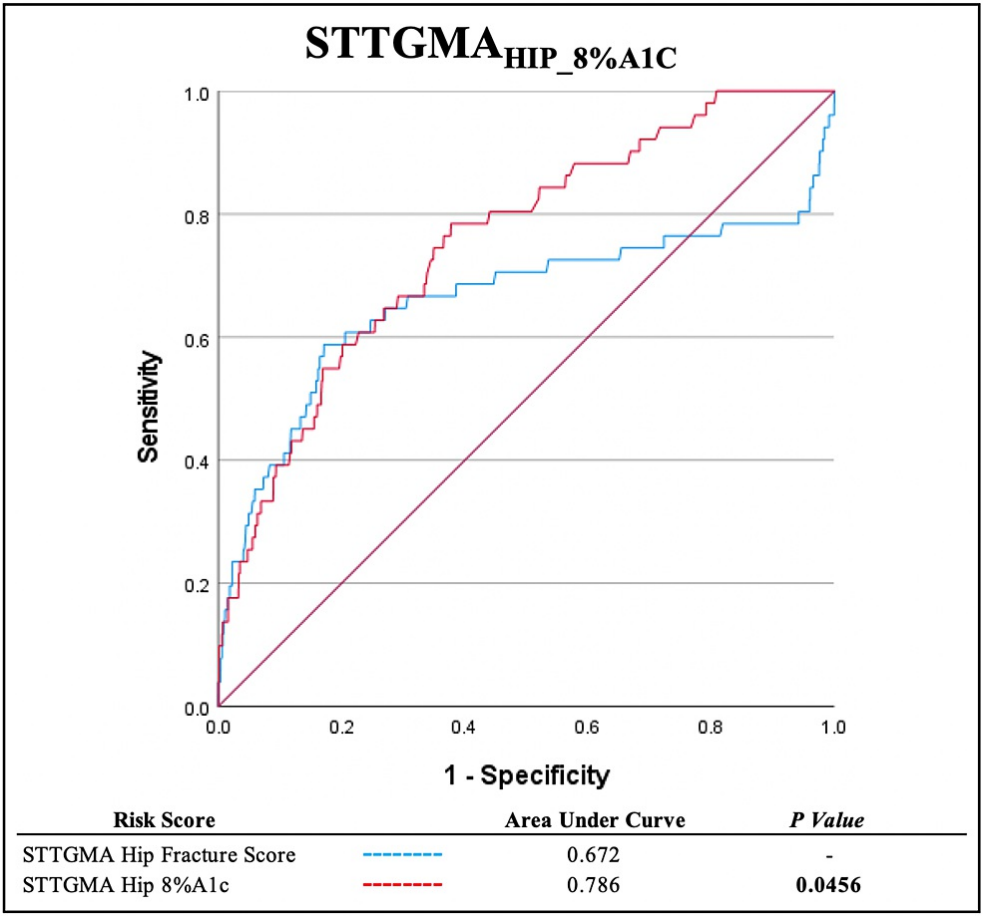
Area under receiver operating curve (AUROC) comparison of STTGMAHIP_FX_SCORE and STTGMAHIP_8%A1c mortality risk scores STTGMA - Score for Trauma Triage in the Geriatric and Middle-Aged; STTGMAHIP_FX_SCORE - Score for Trauma Triage in the Geriatric and Middle-Aged Hip Fracture Score; STTGMAHIP_8%A1c - Score for Trauma Triage in the Geriatric and Middle-Aged Hip 8% Hemoglobin A1c Cutoff Score

A comparison of each model's respective AUROC demonstrated that the STTGMAHIP_8%A1c score significantly improved the predictive capacity for inpatient mortality compared to STTGMAHIP_FX_SCORE (0.786 vs. 0.672, p=0.0456; Figure 1). Regression weighting showed a coefficient of 2.132 for patients who have an A1c value >8%. Other variables in the STTGMAHIP_8%A1c score included age, GCS, AIS head/neck, AIS chest, CCI, ambulatory status, and ASA score. 

Comparison of each risk quartile, determined by each patient's respective STTGMAHIP_8%A1c score, confirmed the stratification tool effectively triaged patient outcomes. Patients stratified to the highest risk cohort were found to have a longer mean length of stay (p<0.001), with higher rates of inpatient (p<0.001) and 30-day mortality (p<0.001), and need for admission to the intensive care unit (p<0.001) as compared to the minimal risk cohort. Patients stratified to the lowest risk quartile experienced the highest rate of home discharge (p<0.001; Table 2).

**Table 2:** Outcome comparison between STTGMAHIP_8%A1c cutoff risk quartiles STTGMA - Score for Trauma Triage in the Geriatric and Middle-Aged; LOS - length of stay; ICU - intensive care unit; STTGMAHIP_8%A1c - Score for Trauma Triage in the Geriatric and Middle-Aged Hip 8% Hemoglobin A1c Cutoff Score

Outcomes	High risk	Moderate risk	Low risk	Minimal risk	Total	p-value
	(100-75%)	(75-50%)	(50-25%)	(25-0%)	n (%)	
STTGMA risk score	>2.20%	2.19%-1.28%	1.27%-0.77%	<0.76%		
N	607	607	602	614	2430	
Major complications (n, %)	115 (18.95%)	74 (12.19%)	50 (8.31%)	32 (5.21%)	271 (11.15%)	<0.001
Minor complications (n, %)	289 (47.61%)	252 (41.52%)	211 (35.05%)	189 (30.78%)	941 (38.72%)	<0.001
Hospital quality measures						
LOS (days, mean ± SD)	7.27 ± 4.58	6.80 ± 5.15	6.23 ± 3.97	5.71 ± 3.74	6.50 ± 4.43	<0.001
Need for ICU	164 (27.02%)	119 (19.60%)	99 (16.45%)	73 (11.89%)	455 (18.72%)	<0.001
Discharged home	88 (14.50%)	76 (12.52%)	151 (25.08%)	260 (42.35%)	575 (23.66%)	<0.001
Readmissions						
Within 30 days	77 (12.69%)	44 (7.25%)	44 (7.31%)	27 (4.40%)	192 (7.90%)	<0.001
Within 90 days	132 (21.75%)	91 (14.99%)	77 (12.79%)	47 (7.65%)	347 (14.28%)	<0.001
Mortality						
Inpatient	31 (5.11%)	10 (1.65%)	7 (1.16%)	3 (0.49%)	51 (2.10%)	<0.001
Within 30 days	66 (10.87%)	27 (4.45%)	18 (2.99%)	7 (1.14%)	118 (4.86%)	<0.001
One year	150 (24.71%)	79 (13.01%)	41 (6.81%)	17 (2.77%)	287 (11.81%)	<0.001

## Discussion

The purpose of this study was to determine whether including a patient's hemoglobin A1c value on admission improved the STTGMA model's predictive capacity to better triage patients treated for hip fracture in the geriatric and middle-aged populations. This study shows the inclusion of a patient's hemoglobin A1c value from the time of admission created a more predictive risk assessment model that can be used to highlight patients at risk for poor outcomes secondary to elevated diabetes lab values. 

This study incorporated a patient's hemoglobin A1c value recorded at the time of admission for their hip fracture into a validated risk assessment tool. Our data demonstrates the addition of this variable improves the predictive capacity for inpatient mortality risk assessment. Some reasons for this may be a patient's A1c being a reflection of their overall health status, with patients that have more poorly controlled diabetes with a higher A1c representing a sicker population at baseline. In addition, diabetes is known to cause end-organ damage over time, especially when poorly controlled; therefore, these patients are likely at higher risk for complications associated with their cardiovascular, respiratory, and renal systems. The correlation between patient A1c levels and mortality rates has been cited in the trauma literature. A comparative retrospective study by Edwards et al. reviewed 2978 patients who sustained a traumatic injury and had a hemoglobin A1c value collected at the time of admission [5]. They found a 50% higher risk of mortality in patients who had A1c values above 5.7% (pre-diabetes) and 6.5% (diabetes). As a result, their study emphasized the utility of recording A1c values and interpreting these values to identify patients at high risk for mortality. A meta-analysis by Fralick et al. reviewed a collection of randomized cardiovascular trials to find that a 0.5% reduction in a patient's A1c value was associated with a lower incidence of cardiovascular events [6]. As cardiovascular complications are common sequelae secondary to poorly controlled diabetes and long-standing hyperglycemia, this highlights the importance of including a patient's hemoglobin A1c value in their management decisions. While not using A1c directly, another comparative study by Rutenberg et al. utilized the Diabetes Complications Severity Index (DCICS) to predict the rates of complications and mortality in patients with diabetes who sustain a hip fracture [15]. The DCIS is a composite score of many variables that includes hemoglobin A1c; therefore, this can be used proxy with higher DCIS correlating with a higher A1c value. Their study then found higher DCIS correlated with a higher one-year mortality rate in patients with diabetes. The findings of these various studies highlight the intimate relationship between a patient's A1c value and their potential outcomes, giving further evidence to the utility and efficacy of the new STTGMAHIP_8%A1c score.

Our study found the usage of a binary variable specifically associated with an A1c threshold (8%) was an effective way to capture a patient's A1c value. While an A1c cutoff of 8% is different from the threshold for poorly controlled that is cited in the literature, our group's past findings of significantly worse outcomes above that level indicate this is an appropriate threshold (Merrell et al.). Likewise, a comparative study by Underwood et al. examined major non-cardiac surgical outcomes to find significant differences in outcomes above an A1c level of 8%, further supporting that the 8% cutoff is a useful clinical cutoff for risk stratification [16]. Finally, another benefit of this dichotomous variable is many patients without a diagnosis of diabetes do not routinely have a hemoglobin A1c value recorded; therefore, the usage of a binary variable may make the model more user-friendly in the acute care setting since patients with no diagnosed history of diabetes and recent glucose readings within normal limits are usually below the 8% cutoff. As a result, this additional variable can be applied to the risk score calculated for all patients regardless of a prior diagnosis of diabetes with routine A1c lab values. 

The inclusion of a patient's admission hemoglobin A1c value in the STTGMA tool continued to provide effective triage, with high-risk patients being categorized into the appropriate risk quartiles. Therefore, the STTGMAHIP_8%A1c tool stratified patients with significant rates of complications, readmission, and mortality into the highest-risk cohort. Identifying these patients early on in their admission can inform the appropriate medical management planning, reduce costs and hopefully improve the patient's perioperative course and long-term outcomes.

This study has several limitations. First, this analysis is a retrospective study that may be confounded by the biases commonly seen in these study designs. Second, as our analysis was derived from an electronic medical record (EMR) review to determine each patient's hemoglobin A1c value at the time of admission, we were unable to document these variables in patients that did not have these lab values within their EMR. However, as our binary variable utilized a threshold of 8%, it is likely safe to assume that patients without any history of diabetes or hyperglycemia were correctly classified as having an A1c below 8%. Third, while the presence of diabetes mellitus may impact various fracture patterns and treatment types (arthroplasty versus operative internal fixation) differently, we sought to include all of these in one cohort for analysis so that the tool may be more generalizable and applicable to any patients that may present with these injuries. Therefore, while the utilization of a different model for each fracture pattern or treatment course may improve each model's performance, this will make the overall usability of the STTGMA tool less user-friendly. Fourth, our study did not take into account which forms of glycemic control different patients were using or whether they had additional forms of end-organ damage secondary to long-standing diabetes. However, as the STTGMA tool accounts for many other comorbidities, including chronic kidney disease and peripheral vascular disease, through the Charlson Comorbidity Index, this limitation is likely minimal. Lastly, future studies may pursue a prospective analysis to compare mortality risks throughout a patient's admission utilizing a patient's glucose as an additional marker for risk stratification.

## Conclusions

The inclusion of this variable, whether a patient's hemoglobin A1c on admission is above or below 8%, as a cutoff score improves the predictive capacity and risk stratification ability of the previously validated STTGMAHIP_FX_SCORE tool. The benefit of this addition is a more effective triage strategy for at-risk patients to predict mortality, adjust care accordingly, and improve outcomes. As diabetes will continue to be another medical challenge for providers to manage following a hip fracture, the new STTGMAHIP_8%A1c tool should utilize this new variable to better highlight at-risk diabetic patients and improve outcomes. It is our hope that this tool may allow for a more interventional response to the associations seen between hemoglobin A1c and outcomes in the geriatric hip fracture population. 
